# Helminthiasis and malaria co-infection among women of reproductive age in a rural setting of Kilifi County, coastal Kenya: A mixed method study

**DOI:** 10.1371/journal.pgph.0003310

**Published:** 2024-06-03

**Authors:** Janet Masaku, Francis Mutuku, Jimmy H. Kihara, Charles Mwandawiro, Collins Okoyo, Henry Kanyi, Joyce Kamau, Lydia Kaduka, Zipporah Ng’ang’a, Victor T. Jeza

**Affiliations:** 1 Eastern and Southern Africa Centre of International Parasite Control (ESACIPAC), Kenya Medical Research Institute (KEMRI), Nairobi, Kenya; 2 Department of Environment and Health Sciences, Technical University of Mombasa, Mombasa, Kenya; 3 Center for Public Health Research, Kenya Medical Research Institute, Nairobi, Kenya; 4 Department of Medical Laboratory Sciences, South Eastern Kenya University, Kitui, Kenya; 5 Department of Medical Sciences, Technical University of Mombasa, Mombasa, Kenya; PLOS: Public Library of Science, UNITED STATES

## Abstract

Soil transmitted helminthiasis (STH), *Schistosoma haematobium* and malaria co-infection lead to increased susceptibility to other infections and poor pregnancy outcomes among women of reproductive age (WRA). This study sought to establish risk factors, burden of co-infection with STH, *S*. *haematobium* and *Plasmodium* sp. among WRA in Kilifi County, Kenya.A mixed method cross-sectional study was conducted on 474 WRA in 2021. Simple random sampling was used to select WRA from four villages in two purposively sampled sub-counties. Study participants were interviewed, and stool samples collected and analysed using Kato-Katz technique for STH. Urine samples were collected for examination of *S*. *haematobium* while malaria microscopic test was done using finger prick blood samples. Further, 15 focus group discussions (FGDs) were conducted with purposively selected WRA and qualitative data analyzed thematically using Nvivo software. Quantitative and qualitative methods were triangulated to comprehensively strengthen the study findings. Prevalence of *S*. *haematobium* was 22.3% (95%CI: 13.5–36.9), any STH 5.2% (95%CI: 1.9–14.3) and malaria 8.3% (95%: 3.8–18.2). Co-infections between any STH and *S*. *haematobium* was 0.8% (95%CI: 0.2–3.2) and between *S*. *haematobium* and malaria 0.8% (95%CI: 0.2–3.1). Multivariable analysis showed increased odds of any STH infections among participants in Rabai Sub-County, (aOR = 9.74; p = 0.026), businesswomen (aOR = 5.25; p<0.001), housewives (aOR = 2.78; p = 0.003), and casual laborers (aOR = 27.03; p<0.001). Qualitative analysis showed that the three parasitic diseases were common and responsible for possible causes of low birth weight, susceptibility to other infections and complications such as infertility and cancer later in life.The study demonstrated that STH, *S*. *haematobium* and malaria are still a public health problem to WRA. Some of the associated risks of infection were geographical location, socio-economic and WASH factors. Hence the need to implement integrated control efforts of the three parasitic infection.

## Introduction

Schistosomiasis is a water-borne parasitic infection, caused by six species of blood flukes of genus *Schistosoma* [1]. It is one of the twenty most common chronic conditions, known as neglected tropical diseases (NTDs), that primarily affect the world’s poorest people [2]. Adult schistosomes invade the human blood vessels and the immune system where they excrete hundreds to thousands of eggs daily [3]. Globally, an estimated 779 million people are at risk of schistosomiasis, more than 230 million are infected, 120 million are symptomatic and 20 million suffer from severe and debilitating forms of schistosomiasis [3].

The disease affects people across all age groups and gender that primarily rely on agriculture for their livelihood [4]. These people are also often co-infected with other parasitic diseases such as malaria and other helminths [5]. Nevertheless, children (aged 10 to 17 years) and women are more affected than men [4, 6]. More so, the effects of infection tend to be greater in women of reproductive age (WRA; 16–49 years) and especially pregnant women [5]. Over the last fifty years, schistosomiasis distribution has reduced in some areas due to successful control but increased in others due to population growth and increased water contact and development projects [7].

In Kenya, schistosomiasis is caused by two main species, *Schistosoma mansoni* which occurs mostly in the western region and *Schistosoma haematobium* also called urogenital schistosomiasis which is predominantly found in the coastal region [8]. The overall prevalence of schistosome infection ranges from 5 to over 65% in communities in Kenya and contributes to significant morbidity [9]. Socio-economic status, human behaviour, environmental factors and demography may be partly responsible for the distribution of this disease [10].

Soil-transmitted helminthiasis (STH) are the most widespread of the NTDs, primarily affecting marginalized populations in low and middle-income countries. Indeed, more than one billion people are currently infected with one, or several, of the common STH species which include *Ascaris lumbricoides*, *Trichuris trichiura*, *Strongyloides stercoralis* and the two hookworm species, *Necator americanus* and *Ancylostoma duodenale* [11]. The infections are mainly caused by ingestion of eggs from contaminated soil (*A*. *lumbricoides*, *S*.*stercoralis* and *T*. *trichiura*) or active penetration of the skin by larvae in the soil (hookworms) [12]. The parasitic disease occurs mainly among rural dwellers in tropical and subtropical countries where poor health awareness, poverty and inadequate sanitation favour the disease transmission [13].

School age children (SAC: 5–14 years) in endemic areas mostly bear the highest burden of these infections due to their frequent exposure to contaminated environment especially when playing, eating unwashed fruits or raw vegetables, or drinking untreated water [14]. To achieve sustained control of STH prevalence, infection intensity, and morbidity, the World Health Organization (WHO) recommends an integrated approach, which includes access to appropriate sanitation, hygiene education, and preventive chemotherapy (PC) [14]. However, most of these approaches are mostly targeted at SAC who have largely benefited through annual mass drug administration (MDAs). Nevertheless, previous studies have shown that adults living in endemic areas of STH are equally infected and act as potential reservoir of these infections if they remain untreated [15].

Malaria is an acute febrile life-threatening disease caused by parasites of the genus *Plasmodium*, which are transmitted to individuals through the bites of infected female *Anopheles* mosquitoes [16]. The parasitic disease is often co-endemic with helminthiasis. Though preventable and curable, it affects the poor leading to poverty through lost productivity, reduction of income, expenses for preventive measures, and deaths [17]. According to the latest World malaria report by WHO, there were an estimated 241 million malaria cases and 627 000 malaria deaths worldwide in 2020 [18]. The heaviest burden continues to be in sub-Saharan Africa which accounts for about 95% of all malaria cases and 96% of all deaths with about 80% of deaths among children under 5 years of age [18].

Over, the last 2 decades, expanded access by WHO-recommended malaria prevention tools and strategies which include effective vector control, use of preventive antimalarial drugs, early diagnosis and treatment to reduce disease transmission and prevent deaths [19]. In Kenya, the Ministry of Health (MOH), estimated malaria in pregnancy (MiP) to be 6.3% among women attending their first antenatal care (ANC) visit in 2019 [20]. Previous studies have reported associations between malaria with anemia in pregnancy which can lead to stillbirths, low birth weights and infant prematurity [21].

Many studies have been done on urogenital schistosomiasis, STH, and malaria in Africa and in Kenya particularly in coastal region where the parasitic diseases are endemic [22, 23]. Nevertheless, much of the effort has been targeting SAC leaving out other vulnerable community members like WRA who might equally be infected. Some of the risk factors for co-infection with the parasitic disease include, exposure to infected mosquitos through bites, eating under cooked food and lack/inadequate water, sanitation and hygiene (WASH). Evidence suggests that WRA, especially pregnant women, are at increased risk of iron deficiency, anemia due in part, to helminths infections [24]. It is notable that the three parasitic diseases lead to double burden in pregnancy which can eventually lead to poor immunity, increased susceptibility to other infections, and poor birth outcomes [17]. Previous studies have shown that deworming resulted in improvements in maternal iron status, increased hemoglobin concentrations, birth weight and perinatal survival [25]. The current study sought to understand the burden of urogenital schistosomiasis, STH and malaria (*Plasmodium* sp.) co-infections and the associated risk factors among WRA in a rural setting of Kilifi County, coastal Kenya.

## Methods

### Ethics statement

Prior to data collection, study protocol was reviewed by Scientific and Ethics Review Unit of Kenya Medical Research Institute (KEMRI) (SERU # KEMRI/SERU/ESACIPAC/3684). The Kenya National Commission for Science, Technology, and Innovation (NACOSTI) certified the study according to the rules and regulations in Kenya. Permission to carry out the study was sought at County level, sub-county, ward and village levels to conduct the study. While written informed consent was sought from the study participants to participate in the study. Also, a written informed consent was obtained from the parent/guardian of each participant under 18 years of age. Study participants who were 15–17 years of age gave assent also to participate in the study.

### Study design and study site

This was a mixed method cross-sectional study, conducted in March/April 2021 in a rural setting of Kilifi County, coastal Kenya. The county is usually hot and dry, with day temperatures of 35°C and 30°C at night. Kilifi County is located north and northeast of Mombasa town at Latitude: 3°37′49″ South and Longitude: 39°50′59″ EastTop of Form with an estimated population of 1,453,787 which covers an area of 12,245.90 km^2^ (4,728.17 sq mi) [26]. There are 298,472 households with an average household size of 4.4 persons and a population density of 116 people per square kilometre [26]. Tourism and fishing in Kilifi County are major economic activities due to its proximity to the Indian ocean. The county has some of the best beaches and historical sites attraction that date back to between the fourteenth and seventeenth century [27]. Cash crop farming of cashew nuts and coconuts is also part of the agricultural activities done in the county with majority of the population practising subsistence farming of crops such as cassava, rice, vegetables, maize and beans [27]. There are few seasonal rivers that transact the county from upper region and several dams constructed for storing water during dry seasons. There are a total of 235 health facilities in the county with one county referral hospital [26].

### Study population and sample size

The target population was WRA drawn from six villages in Magarini and Rabai Sub-Counties. The study villages were selected using simple random sampling in the two sub counties. For quantitative data, the sample size was calculated using the Cochran formula [28]; *n*  =  *Z*^2^*pq*/*e*^2^; where Z is the score for a 5% type 1 error for a normal distribution (Z  =  1.96), p is the assumed prevalence of urogenital schistosomiasis in WRA taken as 50%. This assumed prevalence (50%) was used since there was no similar study conducted in the area previously. The proportion of the population with no infection is represented by the letter q. Using a margin of error (e) of 4.5 and 19% non-response and refusal rate, the final estimated sample size was 474 WRA. Hence, a total of 474 WRA between the ages of 15–50 were enrolled in the study from six randomly selected villages of Burangi, Marikebuni, and Mwangatini in Magarini Sub-County and Boyani, Jimba, and Kanyumbuni in Rabai Sub-County. The study participants were selected using simple random sampling technique as they came to the facility until the final sample size was achieved.

### Community sensitization and data collection

The study team held meetings before the commencement of the study with the county, sub-county leaders and village elders at the community level to sensitize them about the study. During the sensitization period, the study participants were requested to visit a selected health facility in the area on specific dates for data collection. Both quantitative and qualitative methods were used for data collection. Both methods were used to complement each other and further explore between each of the data set. The selected health facilities served as centers for sample collection and conducting interviews. During sample collection (blood, stool and urine), written informed consent forms were signed by all study participants or their guardians for those below the age of eighteen. Data collection for quantitative survey done first, by trained field assistants using android based mobile phones where the pretested questionnaires had been programmed into Open Data Kit (ODK) software [29] Socio-demographic data and water, sanitation and hygiene (WASH) information was obtained during the interviews from all pregnant and non-pregnant women using Swahili language for better understanding by the study participants.

### Qualitative data collection

In the qualitative arm of the study, participants were recruited from those who were participating in the questionnaire survey. However, only WRA who were skipped by the sampling technique of the quantitative method were recruited for qualitative data collection. Fifteen (15) focus group discussions (FGDs) were conducted with the selected WRA. All participants were purposively selected based on their suitability to enhance understanding of the study. Interviews were conducted using interview guides to capture knowledge, perceptions and practices that may be affecting continued infection with helminths. All interviews were conducted in local language (Giriama) or Swahili depending with understanding and preference of the study participants. Before data collection, community meetings were held with village and area chiefs to explain the study’s purpose, procedures, potential risks, and benefits. Before the interviews, the study was explained to the participant, and written informed consent forms were signed by all study participants.

### Parasitological examination

Fresh urine samples were collected from the study participants for examination of *S*. *haematobium* infection using nuclear pore filtration technique [30]. Urine (10 ml) collected from WRA between 10 am and 2 pm was filtered in duplicate through 12.0 μm (13 mm) polycarbonate membrane filters (Sterlitech, Kent, WA, USA) mounted on urine filtration chambers. The membranes were then placed on labelled glass slides and examined using a microscope. Mean egg counts were calculated and expressed as egg counts per 10 ml urine, then categorized as either light (≤ 50 eggs/10 ml urine) or heavy intensity (> 50 eggs/10 ml urine) according to WHO guidelines [31].

Kato-Katz techniques was used to determine the presence or absence of STH ova in the stool sample. In each stool sample collected, duplicate thick smears were prepared from each stool sample using a sieve and a template calibrated to contain 41.7 mg of stool and covered with cellophane strips pre-soaked in glycerol-malachite green solution. The slides were examined using a microscope for STH ova. Egg counts were multiplied by a factor of 24 to obtain the eggs per gram of stool which was categorized as light, moderate, or heavy intensity as per the WHO guidelines [31].

For malaria examination, a drop of venous blood was used to make thin and thick blood smears which were prepared and stained with 2% Giemsa for 30 min, then washed in distilled water for one minute and examined under a microscope by experienced medical laboratory technologist. Thick smears were used to identify parasite densities where malaria parasites were reported against 200 white blood cells counted, while thin blood smears were used to identify the species that is specific of the malaria causing parasites observed under thick blood smears. An experienced medical laboratory technologist examined 10% of all the blood smear slides for quality assurance and quality control. Any discrepancies were repeatedd and reconciled then captured electronically on laboratory reporting data forms that had been programmed onto android smart phones using ODK [32].

### Pregnancy test

Pregnancy test was done using clinical guard pregnancy urine test strips which work by detecting levels of human chorionic gonadotropin (hCG), markers of pregnancy in persons urine. The urine sample in clean containers collected from the study participants was used for the test by dipping half of the absorbent pad for at least 10 seconds. Then wait for five minutes to read the results while the strip lye horizontally on a flat surface. Distinct color bands appear on the Control (C) and Test (T) regions. Consistent color intensity of the bands can vary according to concentration and level of hCG development. The test line is usually slightly weaker in comparison to the control line. The pattern of increasing intensity of the test line is a predictor of pregnancy.

**Qualitative data** collected was audio recorded, transcribed, coded according to pre-determined thematic areas of the study such as knowledge of the parasitic diseases (*S*. *haematobium*, STH and Malaria), causes, symptoms, prevention and control. Others were attitudes towards seeking treatment, perceptions on those mostly affected, perceived expectations when they visit health care facilities and possible practices that lead to repeated infections. The notes of all the transcripts were typed and saved in MS Word spread sheet for coding. The coding process involved a critical review of the transcript and notes to identify emerging themes and sub themes from the data.

### Statistical analysis

#### Quantitative data analysis

Prevalence of malaria, schistosomiasis and STH were calculated and the 95% confidence intervals (CIs) determined using binomial regression model taking into account clustering by villages. Average (arithmetic mean) intensities were determined for schistosomiasis and STH and the associated 95%CIs estimated using negative binomial regression model. We reported the prevalence risk ratio (RR) and the incidence rate ratio (IRR) for binomial and negative binomial regression models respectively. Specifically, prevalence analysis was not carried out for *Ascaris lumbricoides* (STH species) and *Schistosoma mansoni* (Schistosome species), because there were no cases reported.

Risk factor analysis was conducted for malaria, schistosomiasis and STH in a two-step approach. Firstly, univariable analysis was conducted taking into account clustering by villages. Here, estimates were described as odds ratios (OR). Secondly, multivariable analysis was performed in a sequential block-wise approach for each of the outcome of interest, where the selected covariates were included and eliminated one at a time until the most parsimonious model was obtained. Here, adjusted odds ratios (aOR) were obtained by mutually adjusting all minimum generated variables in a multilevel mixed effects logistic regression model at two levels, villages nested within sub counties. Prior to performing multivariable analysis, all covariates that met the inclusion criteria of p<0.05 were investigated for collinearity using pairwise correlation and none of the variables showed significant correlation (r≥0.8). Throughout the analysis, p-value of ≤ 0.05 was considered statistically significant. It is important to note that in this analysis we did not create wealth index variable due to lack of enough measurements related to wealth status of the surveyed participants. All the statistical analyses were carried out using STATA version 15.1 (STATA Corporation, College Station, TX, USA).

### Qualitative data analysis

All notes were imported to NVIVO version 11 software for further processing and data analysis. The software facilitated identification of themes by running queries that allow identification of patterns within the coded data. For specific analyses, simple and advanced coding queries were used to search for words, texts and phrases in the notes. A framework analysis approach based on identified key themes that emerged from the interviews iteratively up to saturation point were adopted and aligned with pre-determined themes [33].

## Results

### Demographics characteristics of the study population

Overall, data was analyzed from 474 WRA in two sub-counties of Kilifi County; 197 (41.6%) from Magarini and 277 (58.4%) from Rabai. For each sub-county, participants were sampled from three villages. The mean age of the participants was 28.9 years (standard deviation (SD): 9.4 years; range 15–49 years) and 107 (22.6%) were aged between 21 and 30 years, while another 107 participants (22.6%) were aged 20 years and below. Further, 92 women (19.4%) were aged between 31 and 40 years, and 77 women (16.2%) were aged between 41 and 50 years (Table 1). Individual and household characteristics have been summarized in Table 1. The level of education varied among the participants with most of them having attained primary level, 305 (64.4%), followed by secondary level (57, (12.0%) and the least was 11 (2.3%) that had post-secondary level. However, 101 (21.3%) had not attended any formal education. Majority of the women were married 350 (73.8%) while 124 (26.2%) were single, widowed, or divorced. The reported average debut age of marriage was 20.0 years (SD: 3.7 years; range: 12–37 years) and 74 women (19.9%) were below 18 years at the time of marriage (Table 1). Some variables had some small proportions of missing values since some participants did not respond to some questions. Further, the consenting process allowed participants not to answer any question they felt uncomfortable with. The variable-specific missing values were not more than 2% of the total response to those variables. All the analyses were done on the complete cases of 474 participants.

**Table 1:** Reported individual household characteristics and the number of individuals positive for STH, *S*. *haematobium* and malaria infections.

Factor	Number examined (%)	STH infection				*S*. *haematobium*	*Malaria*
		*Any STH*	*A*. *lumbroicoides*	Hookworm	*T*. *trichiura*		
Overall	474 (100.0%)	23 (5.2%)	4(0.9%)	20 (4.5%)	3(0.6%)	104(22.3%)	39(8.2%)
**Demographic characteristics**							
***Sub-counties***							
***Magarini sub-county***	197 (41.6%)	2 (1.1%)	0	2 (1.1%)	0	39(20.6%)	30(15.3%)
Burangi village	65 (32.9%)	1 (1.7%)	0	1(1.6%)	0	20(31.7%)	5(7.8%)
Marikebuni village	50 (25.4%)	1 (2.0%)	0	1(2%)	0	0	9(18/0%)
Mwangatini village	82 (41.6%)	0	0	0	0	19(24.6%)	16(19.5%)
***Rabai sub-county***	277 (58.4%)	21 (8.3%)	4(1.5%)	18 (6.9%)	3(1.1%)	65(23.4%)	9(3.2%)
Boyani village	104 (37.6%)	3 (3.2%)	2(2.2%)	3(3.1%)	0	26(25.0%)	3(2.8%)
Jimba village	99 (35.7%)	5 (5.4%)	0	3(3.1%)	2(2.1%)	37(37.3%)	0
Kanyumbuni village	74 (26.7%)	13 (19.4%)	2(2.9%)	12(17.6%)	1(1.4%)	2(2.7%)	6(8.1%)
***Age groups***							
20 years and below	107 (22.6%)	2 (1.9%)	0	2(1.9%)	0	27(25.9%)	13(12.2%)
21–30 years	198 (41.8%)	15 (8.0%)	3(1.6%)	12(6.3%)	3(1.6%)	43(21.7%)	18(9.0%)
31–40 years	92 (19.4%)	2 (2.4%)	0	2(2.3%)	0	18(20.0%)	6(6.5%)
41–50 years	77 (16.2%)	4 (5.7%)	1(1.4%)	4(5.6%)	0	16(21.6%)	2(2.6%)
***Level of education***							
None	101 (21.3%)	4 (4.4%)	0	4(4.4%)	0	25(25.2%)	5(5.0%)
Primary	305 (64.4%)	13 (4.5%)	4(1.3%)	11(3.7%)	2(0.6%)	67(22.4%)	28(9.2%)
Secondary	57 (12.0%)	6 (11.1%)	0	5(9.2%)	1(1.8%)	10(17.5%)	5(8.7%)
Post-secondary	11 (2.3%)	0	0	0	0	2(18.1%)	1(9.0%)
***Average income (Ksh)***							
Below 5000	354 (74.7%)	21 (6.4%)	2(0.6%)	18(5.4%)	3(0.9%)	67(19.2%)	28(7.9%)
Between 5000–10000	95 (20.0%)	2 (2.3%)	2(2.2%)	2(2.2%)	0	29(31.1%)	8(8.4%)
Above 10000	25 (5.3%)	0	0	0	0	8(32%)	3(12.0%)
***Marital status***							
Single/divorced/widowed	124 (26.2%)	7 (5.9%)	2(1.6%)	7(5.8%)	0(0%)	29(23.7%)	13(10.4%)
Married	350 (73.8%)	16 (4.9%)	2(0.6%)	13(3.9%)	3(0.9%)	75(21.8%)	26(7.4%)
***Occupation***							
Farmer	153 (32.3%)	3 (2.1%)	0	1(0.9%)	2(1.3%)	44(29.7%)	16(10.6%)
Business	59 (12.5%)	3 (5.4%)	0	3(5.2%)	0	17(28.8%)	3(5.1%)
Housewife/No job	144 (30.4%)	8 (6.3%)	2(1.5%)	7(5.3%)	1(0.7%)	24(16.6%)	6(4.1%)
Salaried worker	22 (4.6%)	1 (4.8%)	1(4.7%)	1(4.7%)	0	8(36.3%)	0
Casual laborer	25 (5.3%)	3 (12.0%)	0	3(12%)	0	5(20.8%)	2(8.0%)
Others	71 (14.9%)	5 (7.3%)	1(1.4%)	5(7.2%)	0	6(8.7%)	12(16.9%)
***Religion***							
Christian	352 (74.3%)	14 (4.2%)	4(1.2%)	11(3.3%)	3(0.9%)	67(19.4%)	34(9.7%)
Islam	89 (18.8%)	8 (9.9%)	0	8(9.6%)	0	27(30.3%)	1(1.1%)
Non practicing	33 (6.9%)	1 (3.2%)	0	1(3.2%)	0	10(30.3%)	4(12.1%)
**Pregnancy factors**							
***Currently pregnant*** *							
Yes	84 (17.7%)	5 (6.3%)	1(1.2%)	4(5.0%)	1(1.2%)	14(17.0%)	9(10.7%)
No	390 (82.3%)	18 (5.0%)	3(0.8%)	16(4.3%)	2(0.5%)	90(23.4%)	30(7.7%)
***Pregnancy result***							
Positive	111 (23.1%)	6 (5.9%)	1 (1.0%)	5 (5.0%)	1 (1.0%)	17 (15.7%)	12 (10.9%)
Negative	363 (76.8%)	17 (5.0%)	3 (0.9%)	15 (4.3%)	2 (0.6%)	87(24.3%)	27 (7.5%)
***Age when first pregnant***							
<18 years	67 (18.8%)	3 (4.7%)	0	3(4.6%)	0	13(19.7%)	8(11.9%)
18–25 years	262 (73.4%)	10 (4.1%)	3(1.2%)	9(3.6%)	1(0.4%)	58(22.4%)	21(8.1%)
>25 years	28 (7.8%)	5 (19.2%)	1(3.7%)	5(19.2%)	0(0%)	4(14.2%)	0
***Number of pregnancies in lifetime***							
One	55 (15.5%)	5 (9.4%)	2(3.7%)	5(9.4%)	0	15(27.2%)	8(14.8%)
Two	67 (18.8%)	2 (3.2%)	0	2(3.1%)	0	9(13.6%)	3(4.4%)
More than two	234 (65.7%)	11 (5.1%)	2(0.9%)	10(4.6%)	1(0.4%)	51(22.1%)	17(7.3%)
***Number of live births in lifetime***							
None	6 (1.7%)	0	0	0	0	2(33.3%)	2(40.0%)
< 5	233 (65.3%)	13 (6.0%)	3(1.3%)	12(5.4%)	1(0.4%)	46(19.8%)	17(7.3%)
≥ 5	118 (33.1%)	5 (4.6%)	1(0.9%)	5(4.5%)	0	27(23.6%)	10(8.6%)
***Place of receiving ANC***							
Government health facility	72 (85.7%)	4 (6.0%)	1(1.4%)	3(4.4%)	1(1.4%)	11(15.4%)	6(8.3%)
Other facility	6 (7.1%)	1 (16.7%)	0	1(16.6%)	0	1(16.6%)	2(33.3%)
Not attending ANC	6 (7.1%)	0	0	0	0	2(40%)	1(16.6%)
***Ownership of a mosquito net***							
Yes	63 (75.0%)	3 (5.1%)	1(1.6%)	3(5.0%)	0(0%)	11(17.7%)	6(9.5%)
No	21 (25.0%)	2 (10.0%)	0(0%)	1(5.0%)	1(5%)	3(15%)	3(14.2%)
***Frequency of net usage***							
Always	60 (95.2%)	2 (3.6%)	1(1.7%)	2(3.5%)	0	10(16.9%)	6(10.0%)
Sometimes	1 (1.6%)	1 (100%)	0	1(100%)	0	0	0
Never	2 (3.2%)	0	0	0(0%)	0	0	0
***Given iron***							
Yes	34 (40.5%)	2 (6.1%)	0	2(6.0%)	0	5(15.1%)	6(17.6%)
No	50 (59.5%)	3 (6.5%)	1(2.1%)	2(4.3%)	1(2.1%)	9(18.3%)	3(6.0%)
***Taken deworming tablets***							
Yes	60 (71.4%)	2 (3.6%)	1(1.8%)	2(3.6%)	0	10(16.9%)	6(10.0%)
No	24 (28.6%)	3 (12.5%)	0(0%)	2(8.3%)	1(4.1%)	4(17.3%)	3(12.5%)
***Taken antimalarial tablets***							
Yes	55 (65.5%)	3 (5.9%)	1(1.9%)	3(5.8%)	0	8(14.5%)	2(3.6%)
No	29 (34.5%)	2 (7.1%)	0	1(3.5%)	1(3.5%)	6(22.2%)	7(24.1%)
**Individual WASH factors**							
***Handwash after helping child defecate***							
Always	415 (87.6%)	20 (5.2%)	4(1.0%)	18(4.6%)	2(0.5%)	91(22.3%)	33(7.9%)
Sometimes	33 (6.9%)	1 (3.1%)	0	0	1(3.1%)	9(27.2%)	3(9.6%)
Never	26 (5.5%)	2 (8.0%)	0	2(8%)	0(0%)	4(16%)	3(11.5%)
***Handwash before preparing food***							
Always	394 (83.1%)	19 (5.2%)	3(0.8%)	16(4.3%)	3(0.8%)	82(21.0%)	32(8.1%)
Sometimes	40 (8.4%)	3 (7.9%)	0	3(7.6%)	0	10(25.0%)	3(7.6%)
Never	40 (8.4%)	1 (2.8%)	1(2.7%)	1(2.7%)	0	12(32.4%)	4(10.0%)
***Handwash after toilet use***							
Always	462 (97.5%)	23 (5.3%)	4(0.9%)	20(4.5%)	3(0.6%)	100(21.9%)	38(8.2%)
Sometimes	7 (1.5%)	0	0	0	0	3(50.0%)	0
Never	5 (1.1%)	0	0	0	0	1(25.0%)	1(20.0%)
***Anal cleansing material used***							
Toilet paper	36 (7.6%)	1 (2.9%)	0	1(2.8%)	0	6(16.6%)	6(16.6%)
Water	349 (73.6%)	21 (6.4%)	4(1.2%)	18(5.4%)	3(0.9%)	76(21.9%)	22(6.3%)
Leaves/vegetation	74 (15.6%)	1 (1.4%)	0	1(1.4%)	0	16(23.5%)	10(13.5%)
Newspaper	15 (3.2%)	0	0	0	0	6 (23.5%)	1 (6.7%)
**Household WASH factors**							
***Water source for drinking***							
Improved	132 (27.9%)	8 (6.4%)	2(1.6%)	7(5.6%)	1(0.8%)	22(16.6%)	11(8.3%)
Unimproved	342 (72.2%)	15 (4.7%)	2 (0.6%)	13 (4.1%)	2 (0.6%)	82 (24.6%)	28 (8.3%)
***Water source for household***							
Improved	110 (23.2%)	6 (5.6%)	2 (1.9%)	6(5.6%)	0	17(15.4%)	11(10.0%)
Unimproved	364 (76.8%)	17 (5.1%)	2(0.6%)	14 (4.1%)	3 (0.9%)	87 (24.4%)	28 (7.8%)
***Treatment of drinking water***							
Yes	103 (21.7%)	3 (3.0%)	0	2(1.9%)	1(0.9%)	28(27.4%)	8(7.8%)
No	371 (78.3%)	20 (5.8%)	4 (1.2%)	18 (5.2%)	2 (0.6%)	76 (20.9%)	31 (8.4%)
***Methods for water treatment***							
Boiling	20 (19.4%)	0	0	0	0	5(25.0%)	0
Chlorine/Bleach	62 (60.2%)	3 (5.0%)	0	2(3.3%)	1(1.6%)	14(22.5%)	8(13.1%)
Others	21 (20.4%)	0	0	0(0%)	0(0%)	9(45.0%)	0
**Type of toilet facility**							
Improved	143 (30.2%)	7 (5.2%)	2(1.4%)	6(4.4%)	1(0.7%)	29(20.4%)	12(8.3%)
Unimproved	225 (47.5%)	14 (6.7%)	2 (0.9%)	12 (5.7%)	2 (0.9%)	53 (23.7%)	13 (5.9%)
No facility/bush	106 (22.4%)	2 (2.0%)	0	2 (2.0%)	0	22 (22.0%)	14 (13.2%)
**Household assets**							
Electricity	66 (13.9%)	5 (7.9%)	2(3.1%)	5(7.9%)	0	10(15.1%)	7(10.6%)
Radio	173 (36.5%)	12 (7.4%)	3(1.8%)	11(6.7%)	1(0.6%)	38(22.3%)	17(9.8%)
Television	376 (79.3%)	3 (3.3%)	2(2.2%)	3(3.2%)	0	17(17.3%)	8(8.1%)
Mobile phone	388 (81.9%)	16 (4.4%)	2(0.5%)	14(3.8%)	2(0.5%)	86(22.6%)	34(8.8%)
Bank account	41 (8.7%)	1 (2.7%)	0	1(2.6%)	0	7(18.4%)	4(10.0%)
Agricultural land	416 (87.8%)	18 (4.7%)	3(0.7%)	16(4.0%)	2(0.5%)	96(23.53%)	36(8.7%)
Cows and goats	227 (47.9%)	11 (5.2%)	3(1.4%)	10(4.6%)	1(0.4%)	50(22.3%)	21(9.3%)
Chicken and ducks	364 (76.8%)	18 (5.3%)	3(0.8%)	15(4.3%)	3(0.8%)	82(22.8%)	26(7.2%)

*Enrolled in the study while already knowing their pregnancy status

The average monthly income of the households was KES 3,222 (SD: 4671; range: 45–40,000). Majority of the participants, 354 (74.7%) had an average monthly income of below KES 5,000, followed by 95 (20.0%) whose average income was between KES 5,000 and 10,000 while 25 (5.3%) had an average income of above KES 10,000. Some household possessions reported among the participants were electricity connection (66 (13.9%)), radio (173 (36.5%)), television (98 (20.7%)), mobile phone (388 (81.9%)), and agricultural land (416 (87.8%)). The most reported occupation was farming 153 (32.3%), business 59 (12.5%), casual laborer 25 (5.3%) and salaried worker 22 (4.6%). Also, 144 (30.4%) women were housewives/had no economic occupation (Table 1).

### Pregnancy among the study participants

Of the participants surveyed, 357 (75.3%) reported to having had previous pregnancies with the average age of first pregnancy being 20.3 years (SD: 3.8 years; range:11–37). The average number of previous pregnancies among the participants was 4.1 (SD: 2.5; range: 1–13) with average live births of 3.6 (SD: 2.3 live births; range:0–11). Out of all participants, 84 (17.7%) reported to being pregnant at the time of the survey. However, the pregnancy test administered to all participants at the time of survey showed that only 111 (23.1%) women were positive (pregnant) (Table 1). Those participants who tested positive with the pregnancy test where informed of their results by the facility health worker who had been recruited by the study team to be a liaison between the research team and study participants.

### Socio-demographic characteristics of the FGD participants

There were 15 focus group discussions (FGDs) that were conducted and a total of 163 females participated. Participants were from 6 sites namely Marekebuni (29 females), Mwangatini (33 females), Kanyumbuni (31 females), Jimba (26 females), Burangi (24 females) and Boyani (20 females). The average age of the female participants was 27.7 years (Standard deviation (SD):8.3 years and range: 15–49 years). The education level of the participants was primary level (112 females; 69.1%), and secondary level (20; 12.3%) while one participant had not attained any formal education level. By age categories, most participants were aged between 21 and 30 years (75; 46.3%), below 20 years (36; 22.2%), between 31 and 40 years (35; 21.6%) and above 40 years (16 females; 9.9%) (Table 2).

**Table 2:** Sociodemographic characteristics of qualitative study participants.

Description	Frequency (n)	Percentage (%)
Gender		
Female	163	100.0%
Site		
Boyani	20	12.3%
Burangi	24	14.7%
Chilodi	26	16.0%
Kanyumbuni	31	19.0%
Marekebuni	29	17.8%
Mwangatini	33	20.2%
n	163	100.0%
Age categories		
20 and below	36	22.2%
21–30 years	75	46.3%
31-40years	35	21.6%
Above 40 years	16	9.9%
n	162	100.0%
Education level		
No education	1	0.8%
Primary	112	84.2%
Secondary	20	15.0%
n	133	100.0%
Occupation		
Business	24	16.0%
Casual labourer	2	1.3%
Employed	1	0.7%
Farmer	27	18.0%
Housewife	70	46.7%
Student	26	17.3%
n	150	100.0%

### STH prevalence and mean intensity among the study participants

The overall prevalence of any STH infection was 5.2% (95%CI: 1.9–14.3) with the highest prevalence recorded in Rabai Sub-County at 8.3% (95%CI: 2.9–23.8) and the least in Magarini Sub-County at 1.1% (95%CI: 0.3–3.6). For the STH species, hookworm infection was highest at 4.5% (95%CI: 1.5–13.1), followed by *A*. *lumbricoides* infection at 0.9% (95%CI: 0.3–2.9) and *T*. *trichiura* infection at 0.7% (95%CI: 0.2–2.4) (Table 3). For each STH species, the mean (arithmetic) intensity (epg) was highest for hookworm infections 14 epg (95%CI: 5–40) and the least for *A*. *lumbricoides* infections 2 epg (95%CI: 0–12) while *T*. *trichiura* had 0 epg. All the infections were categorized as light intensity. Hookworm intensity, expressed in eggs per gram (epg) of feces, was highest for Rabai Sub-County at 22 epg (95%CI: 7–68) and least in Magarini sub-County at 3 epg (95%CI: 1–17). Similarly, *A*. *lumbricoides* intensity was highest in Rabai Sub-County at 4 epg (95%CI: 1–24) (Table 3). Positive cases were treated by the facility pharmacist or nurse in charge using albendazole.

**Table 3:** Prevalence and mean intensity of STH and *S*. *haematobium* infections by sub-county and village.

Villages	Number examined (%)	STH infection					*S*. *haematobium* (n = 104)
		Any STH (n = 23)	*A*. *lumbricoides* (n = 4)	Hookworms (n = 20)	*T*. *trichiura* (n = 3)		
***Prevalence of infection*, *%(95%CI)***							
**Magarini sub-county**	**197 (41.6%)**	**1.1 (0.3–3.6)**	**0**	**1.1 (0.3–3.6)**	**0**	**20.6 (9.5–45.0)**	
Burangi village	65 (32.9%)	1.7 (0.2–11.6)	0	1.7 (0.2–11.6)	0	31.7 (22.1–45.6)	
Marikebuni village	50 (25.4%)	2.0 (0.3–13.9)	0	2.0 (0.3–13.9)	0	0	
Mwangatini village	82 (41.6%)	0	0	0	0	24.7 (16.7–36.5)	
**Rabai sub-county**	**277 (58.4%)**	**8.3 (2.9–23.8)**	**1.6 (0.5–4.6)**	**6.9 (2.1–22.8)**	**1.2 (0.4–3.6)**	**23.5 (10.9–50.4)**	
Boyani village	104 (37.6%)	3.2 (1.0–9.7)	2.1 (0.5–8.3)	3.2 (1.0–9.6)	0	25.0 (17.9–34.9)	
Jimba village	99 (73.3%)	5.3 (2.3–12.6)	0	3.2 (1.0–9.6)	2.1 (0.5–8.3)	37.4 (28.9–48.2)	
Kanyumbuni village	74 (26.7%)	19.4 (11.9–31.6)	2.9 (0.8–11.5)	17.6 (10.6–29.5)	1.4 (0.2–10.1)	2.7 (0.7–10.6)	
**Overall prevalence**	**474 (100.0%)**	**5.2 (1.9–14.3)**	**0.9 (0.3–2.9)**	**4.5 (1.5–13.1)**	**0.7 (0.2–2.4)**	**22.3 (13.5–36.9)**	
Mean* ***intensity of infection*, *epg (95%CI)***							
**Magarini sub-county**	**197 (41.6%)**	**-**	**0**	**3 (1–17)**	**0**	**20 (9–43)**	
Burangi village	65 (32.9%)	-	0	2 (0–263)	0	31 (11–83)	
Marikebuni village	50 (25.4%)	-	0	10 (0–2573)	0	0	
Mwangatini village	82 (41.6%)	0	0	0	0	23 (8–65)	
**Rabai sub-county**	**277 (58.4%)**	**-**	**4 (1–24)**	**22 (7–68)**	**0 (0–1)**	**34 (14–83)**	
Boyani village	104 (37.6%)	-	1 (0–26)	7 (0–154)	0	57 (22–150)	
Jimba village	99 (73.3%)	-	0	14 (1–347)	0 (0–7)	34 (16–72)	
Kanyumbuni village	74 (26.7%)	-	12 (0–610)	54 (13–229)	0 (0–8)	0 (0–2)	
Overall mean* **intensity**	**474 (100.0%)**	**-**	**2 (0–12)**	**14 (5–40)**	**0**	**28 (15–54)**	

*Intensity was computed using arithmetic mean

In the qualitative analysis, intestinal worms were mentioned as one of the common diseases in the area. The local name was *“minyolo or Kipicho”*. However, study participants seemed not to differentiate between intestinal worms and *S*. *haematobium* probably due to the use of the Swahili name ‘‘*minyoo*”. Other common diseases mentioned were ring worms, high blood pressure and malaria. Some of the quotes are listed below; -

*P3; Bilharzia and malaria because we have stagnant water and also the children play in it*, *you will see the children with skin rushes*

P1; The common one is bilharzia, running nose for kids

P2; We have chest pain, coughing and another disease called ‘Pumu’ which means pneumonia

P7; High blood pressure has become common because of the anxiety that has been created by the Covid-19, so you will experience a headache which does not go away

*P4; we have bilharzia we call it “Kipicho” or minyolo (in chorus)*

### *Schistosoma haematobium* prevalence and mean intensity among the study participants

The overall prevalence of *S*. *haematobium* was 22.3% (95%CI: 13.5–36.9) with highest prevalence of 23.5% (95%CI: 10.9–50.4) in Rabai sub-County and 20.6% (95%CI: 9.5–45.0) for Magarini sub-County (Table 3). The overall mean (arithmetic) intensity (eggs/10ml of urine) was 28 eggs/10ml (95%CI: 15–54). For *S*. *haematobium* infections, 15.9% were categorized as light infections (< 50 eggs/10ml) and 6.4% were categorized as heavy infections (≥ 50eggs/10ml). Participants having light and heavy infection intensities from Rabai sub-county were 17.3% and 6.1% respectively while from Magarini sub-county were 13.8% and 6.9% respectively. Prevalence of *S*. *haematobium* was highest in women aged ≤ 20 years 25.9% (95%CI: 17.6–38.3), followed by those aged 21–30 years 21.7% (95%CI: 11.5–41.2), those aged 41–50 years 21.6% (11.8–39.7) and least in those 31–40 years 20.0% (95%CI: 12.7–31.5). Among those who were currently pregnant (i.e., 27/470; 5.7%), the prevalence of *S*. *haematobium* was 11.1% (95%CI: 2.0–61.7) while prevalence among the non-pregnant was 23.2% (95%CI: 14.1–38.3). The intensities of infections among the pregnant women categorized as heavy infections were 66.7% (2/3) while light infections were 33.3% (1/3). All the positive cases were treated with praziquantel based on weight/kilogram.

For qualitative analysis, schistosomiasis also known as bilharzia was mentioned as one of the common diseases found in the area. Study participants felt that those at risk of getting infection with schistosomiasis were children and women due to increased water contact while playing and swimming in water bodies. While for women, they were likely to get infected when in contact with infested water bodies during domestic chores like fetching water, washing dishes, and clothes as listed in some of the quotes below; -

*P10; “It is more in the children especially in our village*, *we have like 5 children who have bilharzia*, *and this is because they like playing in the dirty water*. *Even if they are prohibited from going they will go after their parents leave*. *When you take them to the hospital and they are having a running nose you will also be told they have bilharzia”*

P3; “Our children like going to play in the rivers very much and if there are some of them with bilharzia they will urinate in the water and transmit to the others”.

P7; “I think it is the children but even us the women we are at risk because we fetch water from the stagnant wells, when fetching the water hygiene is not observed because we get in the water with our bare feet. So, we step on the water every day, so we are at risk as well”.

*P2; “The risk is across board because where the children swim it is the same place we fetch water*, *not every time we boil water for bathing especially now that it is sunny I will use the cold water which is not clean which leaves me at the risk of getting bilharzia*. *So*, *all of us we are at risk*, *if you get the disease accept and take drugs then you will be fine”*.

Majority of the study participants had knowledge on the signs and symptoms of the parasitic diseases. Some of the mentioned signs were itching when passing urine, blood in the urine and some discomfort in the stomach. Some of the quotes are listed below; -

*P5; “Some of the symptom will be itching when you pass urine and you don’t know what the problem is*, *so at that point is when you will visit the hospital to know what the problem is then you get treatment”*

P7; “If you see the symptom of blood, it will make you to go the hospital and explain to the doctor what you are feeling then you will be treated and get cured”

*P6; “You feel that your stomach is tight*, *when you urinate there is irritation*, *I had these symptoms and I went to the hospital at Mazeras and got tested and given drugs but up to now I still have the same problem*, *so I don’t know what the problem could be*.

In addition, some of the study participants new that bilharzia infection can expose someone to other infections. When asked the possible effects of bilharzia in WRA, surprisingly some of the study participants had knowledge on the likelihood to get other complications later in life. However, they indicated that health workers in the health facilities have been educating them on the possible effects of the disease if left untreated as the quotes listed below;

*P7; “If you get bilharzia and you are not treated early it will be easy for you to contract those other diseases”*.

P1; “Yes, because if the disease is not treated early, it will bring other complications which can lead to infertility, also can lead to getting cancer, ulcers because it was not treated early”

*P7; “If the disease has been left in the body for a long time it will bring ulcers*, *cancer*, *the uterus will be affected by the worms”*.

When asked the possible effect of bilharzia infection to expectant mothers, most of them knew the disease can affect the birth outcomes including underweight as listed in the quotes below;

*P3; “Yes*, *bilharzia can affect a pregnant mother*, *the only problem is the baby will be born underweight but not with bilharzia”*.

P9; **“**When a pregnant woman has bilharzia, it will affect the baby by giving birth to a weak baby, the baby can have one and half kilograms instead of two and half kilos”.

*P9; Okay what I think is*, *bilharzia is just like any other disease*, *so if the health of the mother will not be okay*, *the mother’s weight is not good the same will happen to the baby”*.

### Malaria prevalence and bed net usage among the study participants

The overall prevalence of malaria was 8.3% (95%: 3.8–18.2) and the highest infections occurring in Magarini sub-County 15.3% (95%CI: 9.4–24.9) compared to Rabai sub-County 3.3% (95%CI: 0.9–11.8) (Table 4). By age groups, the prevalence was highest among participants aged ≤ 20 years 12.3% (95%CI: 6.2–24.4), followed by those aged 21–30 years 9.1% (95%CI: 3.4–24.4), those between 31–40 years 6.6% (95%CI: 2.2–20.2) and least among those 41–50 years 2.6% (95%CI: 0.3–24.3). Among pregnant women (n = 27), prevalence of malaria was higher as compared to non-pregnant women (n = 443), 11.1% (2.0–61.7) and 7.9% (95%CI: 3.4–18.8) respectively. Among those who reported being pregnant (n = 84), bed net ownership was high 63 (75.0%) and the prevalence of malaria was 9.5% (95%CI: 1.6–56.4) (Table 4). All the positive cases were treated in the health facility.

**Table 4:** Prevalence of malaria infections by sub-county and village.

Categories	Number examined; N (%)	Prevalence %(95%CI) (n = 39)
**Overall**	**474 (100.0%)**	**8.3 (3.8–18.2)**
**Magarini sub-county**	**197 (41.6%)**	**15.3 (9.4–24.9)**
Burangi village	65 (32.9%)	7.8 (3.4–18.1)
Marikebuni village	50 (25.4%)	18.0 (9.9–32.5)
Mwangatini village	82 (41.6%)	19.5 (12.6–30.3)
**Rabai sub-county**	**277 (58.4%)**	**3.3 (0.9–11.8)**
Boyani village	104 (37.6%)	2.9 (0.9–8.8)
Jimba village	99 (73.3%)	0
Kanyumbuni village	74 (26.7%)	8.1 (3.8–17.5)
**By age group**		
20 years and below	107 (22.6%)	12.3 (6.2–24.4)
21–30 years	198 (41.8%)	9.1 (3.4–24.4)
31–40 years	92 (19.4%)	6.6 (2.2–20.2)
41–50 years	77 (16.2%)	2.6 (0.3–24.3)
**Bednet ownership**		
Yes	63 (75.0%)	9.5 (1.6–56.4)
No	21 (25.0%)	14.3 (3.9–51.2)
**Pregnancy result**		
Positive	111 (23.1%)	11.0 (2.8–42.1)
Negative	363 (76.8%)	7.5 (3.4–16.5)

In the qualitative analysis, malaria infection was mentioned by the study participants as one of the major common diseases found in the area. This notwithstanding, it is worth to note that other diseases like typhoid and amoebiasis may at times present with similar symptoms which can be confused to be malaria infection by the community members. Below are some of the quotes listed from the study participants.

*P10; “Where I come from*, *it is malaria which is common especially for the children when they have a running nose it will be accompanied by malaria”*.

*P6; “I also wanted to say malaria but nowadays there are some children who have skin infection*, *they will scratch their bodies and some rashes will appear”*.

### Co-infection with *Schistosoma haematobium*, STH and malaria infections

The occurrence of co-infection was low and was recorded between any STH and *S*. *haematobium* at 0.8% (95%CI: 0.2–3.2) and between *S*. *haematobium* and malaria at 0.8% (95%CI: 0.2–3.1). There were no co-infections between any STHs and malaria infections. Results of Rabai sub-county showed that co-infection between any STHs and *S*. *haematobium* was 1.4% (95%CI: 0.4–5.0) while this co-infection was not observed in Magarini Sub-County. However, co-infection between *S*. *haematobium* and malaria was observed in Magarini at 2.0% (95%CI: 0.9–4.3) while no co-infection was observed in Rabai sub-county. Among pregnant women, there were no co-infections between any STHs and *S*. *haematobium* species while among non-pregnant women prevalence was 0.9% (95%CI: 0.2–3.6). Similarly, there was no co-infection between *S*. *haematobium* and malaria infections among pregnant women whereas co-infection prevalence was 0.9% (95%CI: 0.3–3.2) among non-pregnant women (Table 5).

**Table 5:** Univariable analysis of factors associated with STH, *S*. *haematobium* or malaria.

Factor	Number examined (%)	Univariable logistic regression [OR; p-value]			
		STH combined (n = 23)	Hookworm (n = 20)	*S*. *haematobium* (n = 104)	Malaria (n = 39)
**Demographic characteristics**					
***Sub-counties***					
Magarini sub-county	197 (41.6%)	**Reference**	**Reference**	**Reference**	**Reference**
Rabai sub-county	277 (58.4%)	8.43 (1.85–38.46), p = 0.006*	7.01 (1.43–34.51), p = 0.017*	1.18 (0.34–4.13), p = 0.797	0.19 (0.05–0.68), p = 0.011*
***Age groups***					
20 years and below	107 (22.6%)	**Reference**	**Reference**	**Reference**	**Reference**
21–30 years	198 (41.8%)	4.40 (0.90–21.64), p = 0.068	3.48 (0.60–20.28), p = 0.166	0.79 (0.49–1.29), p = 0.347	0.72 (0.20–2.54), p = 0.605
31–40 years	92 (19.4%)	1.25 (0.09–17.92), p = 0.871	1.24 (0.09–17.37), p = 0.871	0.71 (0.44–1.15), p = 0.163	0.50 (0.26–1.00), p = 0.049*
41–50 years	77 (16.2%)	3.06 (0.32–28.84), p = 0.328	3.04 (0.32–28.64), p = 0.330	0.79 (0.52–1.19), p = 0.257	0.19 (0.02–2.49), p = 0.208
***Level of education***					
None	101 (21.3%)	**Reference**	**Reference**	**Reference**	**Reference**
Primary	305 (64.4%)	1.01 (0.41–2.52), p = 0.978	0.84 (0.29–2.40), p = 0.743	0.85 (0.57–1.27), p = 0.439	1.91 (1.08–3.38), p = 0.027*
Secondary	57 (12.0%)	2.69 (1.09–6.64), p = 0.032*	2.19 (1.15–4.19), p = 0.017*	0.63 (0.35–1.13), p = 0.119	1.81 (0.39–8.47), p = 0.453
Post-secondary	11 (2.3%)	-	-	0.66 (0.24–1.83), p = 0.422	1.88 (0.54–6.49), p = 0.318
***Average income (Ksh)***					
Below 5000	354 (74.7%)	**Reference**	**Reference**	**Reference**	**Reference**
Between 5000–10000	95 (20.0%)	0.34 (0.03–4.29), p = 0.402	0.40 (0.03–5.15), p = 0.484	1.90 (1.26–2.86), p = 0.002*	1.06 (0.62–1.82), p = 0.830
Above 10000	25 (5.3%)	-	-	1.97 (0.56–6.96), p = 0.290	1.57 (1.10–2.26), p = 0.014*
***Marital status***					
Single/divorced/widowed	124 (26.2%)	**Reference**	**Reference**	**Reference**	**Reference**
Married	350 (73.8%)	0.82 (0.24–2.84), p = 0.756	0.66 (0.14–3.02), p = 0.593	0.89 (0.49–1.65), p = 0.719	0.69 (0.24–1.98), p = 0.491
***Occupation***					
Farmer	153 (32.3%)	**Reference**	**Reference**	**Reference**	**Reference**
Business	59 (12.5%)	2.66 (0.44–16.08), p = 0.286	8.00 (0.53–119.76), p = 0.132	0.96 (0.48–1.89), p = 0.899	0.46 (0.13–1.63), p = 0.228
Housewife/No job	144 (30.4%)	3.13 (0.91–10.80), p = 0.070	8.19 (0.84–80.37), p = 0.071	0.47 (0.25–0.90), p = 0.023*	0.37 (0.12–1.10), p = 0.073
Salaried worker	22 (4.6%)	2.35 (0.42–13.05), p = 0.329	7.20 (0.98–52.64), p = 0.052	1.35 (0.97–1.87), p = 0.072	-
Casual laborer	25 (5.3%)	6.41 (1.04–39.42), p = 0.045*	19.64 (1.24–311.55), p = 0.035*	0.62 (0.18–2.21), p = 0.462	0.73 (0.29–1.87), p = 0.517
Others	71 (14.9%)	3.67 (1.78–7.57), p<0.001*	11.25 (3.04–41.67), p<0.001*	0.23 (0.08–0.65), p = 0.006*	1.72 (0.71–4.12), p = 0.227
***Religion***					
Christian	352 (74.3%)	Reference	Reference	Reference	Reference
Islam	89 (18.8%)	2.48 (1.06–5.80), p = 0.036*	3.12 (1.15–8.49), p = 0.026*	1.80 (1.04–3.11), p = 0.035*	0.11 (0.02–0.53), p = 0.006*
Non practicing	33 (6.9%)	0.75 (0.06–9.02), p = 0.824	0.98 (0.06–16.50), p = 0.986	1.80 (0.81–3.99), p = 0.149	1.28 (0.59–2.77), p = 0.527
**Pregnancy factors**					
***Currently pregnant***					
Yes	84 (17.7%)	1.30 (0.33–5.19), p = 0.711	1.17 (0.31–4.41), p = 0.813	0.67 (0.30–1.52), p = 0.341	1.43 (0.24–8.37), p = 0.693
No	390 (82.3%)	**Reference**	**Reference**	**Reference**	**Reference**
***Tested pregnant at the point of enrolment*** ^***$***^					
Positive	111 (23.1%)	**Reference**	**Reference**	**Reference**	**Reference**
Negative	363 (76.8%)	0.83 (0.24–2.86), p = 0.765	0.87 (0.27–2.80), p = 0.816	1.72 (0.62–4.74), p = 0.296	0.66 (0.15–2.86), p = 0.579
18–25 years	262 (73.4%)	0.88 (0.38–2.03), p = 0.759	0.79 (0.33–1.89), p = 0.598	1.18 (0.72–1.94), p = 0.508	0.65 (0.18–2.34), p = 0.511
>25 years	28 (7.8%)	**4.84 (1.82–12.89), p = 0.002** *	**4.92 (1.87–12.91), p = 0.001** *	0.68 (0.25–1.86), p = 0.453	-
***Number of pregnancies in lifetime***					
One	55 (15.5%)	**Reference**	**Reference**	**Reference**	**Reference**
Two	67 (18.8%)	0.31 (0.10–0.97), p = 0.044*	0.31 (0.10–0.97), p = 0.044*	0.42 (0.18–0.99), p = 0.047*	0.27 (0.08–0.91), p = 0.035*
More than two	234 (65.7%)	0.52 (0.24–1.12), p = 0.094	0.46 (0.22–0.96), p = 0.039	0.76 (0.48–1.21), p = 0.247	0.45 (0.26–0.80), p = 0.007*
***Number of live births in lifetime***					
None	6 (1.7%)	**Reference**	**Reference**	**Reference**	**Reference**
< 5	233 (65.3%)	-	-	2.02 (0.26–15.80), p = 0.502	8.47 (1.29–55.59), p = 0.026*
≥ 5	118 (33.1%)	0.75 (0.22–2.53), p = 0.648	0.83 (0.23–3.06), p = 0.784	1.25 (0.81–1.95), p = 0.311	1.20 (0.80–1.80), p = 0.383
***Place of receiving ANC***					
Government health facility	72 (85.7%)	**Reference**	**Reference**	**Reference**	**Reference**
Other facility	6 (7.1%)	3.15 (0.34–29.01), p = 0.311	4.27 (0.42–42.87), p = 0.218	1.09 (0.33–3.66), p = 0.888	5.50 (1.02–29.80), p = 0.048*
Not attending ANC	6 (7.1%)	-	-	3.64 (1.30–10.13), p = 0.014*	2.20 (0.38–12.80), p = 0.380
***Ownership of a mosquito net***					
Yes	63 (75.0%)	0.48 (0.06–3.85), p = 0.492	1.02 (0.09–12.11), p = 0.989	1.22 (0.27–5.56), p = 0.795	0.63 (0.22–1.82), p = 0.396
No	21 (25.0%)	**Reference**	**Reference**	**Reference**	**Reference**
***Frequency of net usage***					
Always	60 (95.2%)	**Reference**	**Reference**	**Reference**	**Reference**
Sometimes	1 (1.6%)	-	-	-	-
Never	2 (3.2%)	-	-	4.90 (0.21–111.96), p = 0.319	-
***Given iron***					
Yes	34 (40.5%)	**Reference**	**Reference**	**Reference**	**Reference**
No	50 (59.5%)	1.08 (0.13–8.86), p = 0.942	0.70 (0.08–5.90), p = 0.747	1.26 (0.55–2.90), p = 0.586	0.29 (0.13–0.70), p = 0.004
***Taken deworming tablets***					
Yes	60 (71.4%)	0.26 (0.05–1.48), p = 0.131	0.42 (0.06–2.81), p = 0.367	0.97 (0.32–2.92), p = 0.956	0.78 (0.28–2.19), p = 0.635
No	24 (28.6%)	**Reference**	**Reference**	**Reference**	**Reference**
***Taken antimalarial tablets***					
Yes	55 (65.5%)	0.81 (0.08–8.04), p = 0.859	1.69 (0.11–25.27), p = 0.705	0.59 (0.40–0.88), p = 0.010*	0.12 (0.07–0.20), p<0.001*
No	29 (34.5%)	**Reference**	**Reference**	**Reference**	**Reference**
**Individual WASH factors**					
***Handwash after helping child defecate***					
Always	415 (87.6%)	**Reference**	**Reference**	**Reference**	**Reference**
Sometimes	33 (6.9%)	0.59 (0.09–4.02), p = 0.590	-	1.31 (0.57–3.01), p = 0.531	1.24 (0.24–6.26), p = 0.797
Never	26 (5.5%)	1.59 (1.19–13.20), p = 0.667	1.80 (0.20–16.55), p = 0.605	0.66 (0.37–1.18), p = 0.162	1.51 (0.69–3.30), p = 0.306
***Handwash before preparing food***					
Always	394 (83.1%)	**Reference**	**Reference**	**Reference**	**Reference**
Sometimes	40 (8.4%)	1.58 (0.88–2.83), p = 0.124	1.85 (1.11–3.09), p = 0.019*	1.25 (0.86–1.81), p = 0.242	0.94 (0.30–2.89), p = 0.911
Never	40 (8.4%)	0.52 (0.05–5.93), p = 0.603	0.62 (0.05–6.94), p = 0.695	1.80 (0.67–4.81), p = 0.243	1.25 (0.59–2.64), p = 0.559
***Handwash after toilet use***					
Always	462 (97.5%)	**Reference**	**Reference**	**Reference**	**Reference**
Sometimes	7 (1.5%)	-	-	3.56 (0.87–14.60), p = 0.078	**-**
Never	5 (1.1%)	-	-	1.19 (0.38–3.74), p = 0.770	2.77 (0.35–21.66), p = 0.332
***Anal cleansing material used***					
Toilet paper	36 (7.6%)	**Reference**	**Reference**	**Reference**	**Reference**
Water	349 (73.6%)	2.34 (0.29–18.79), p = 0.424	1.97 (0.23–16.55), p = 0.533	1.40 (0.71–2.76), p = 0.327	0.34 (0.13–0.86), p = 0.022*
Newspaper	74 (15.6%)	-	-	3.33 (0.95–11.66), p = 0.060	0.36 (0.03–4.94), p = 0.442
Leaves/vegetation	15 (3.2%)	0.49 (0.03–8.26), p = 0.623	0.49 (0.03–7.93), p = 0.612	1.54 (0.90–2.63), p = 0.114	0.78 (0.51–1.20), p = 0.260
**Household WASH factors**					
***Water source for drinking***					
Improved	132 (27.9%)	1.36 (0.55–3.38), p = 0.501	1.39 (0.61–3.21), p = 0.435	0.61 (0.23–1.63), p = 0.328	1.01 (0.21–4.96), p = 0.990
Unimproved	342 (72.2%)	**Reference**	**Reference**	**Reference**	**Reference**
***Water source for household use***					
Improved	110 (23.2%)	1.11 (0.36–3.42), p = 0.849	1.38 (0.41–4.64), p = 0.599	0.57 (0.16–2.05), p = 0.385	1.32 (0.30–5.77), p = 0.711
Unimproved	364 (76.8%)	**Reference**	**Reference**	**Reference**	**Reference**
***Treatment of drinking water***					
Yes	103 (21.7%)	0.50 (0.23–1.08), p = 0.077	0.37 (0.09–1.51), p = 0.165	1.43 (0.83–2.48), p = 0.197	0.93 (0.29–3.00), p = 0.901
No	371 (78.3%)	**Reference**	**Reference**	**Reference**	**Reference**
***Methods for water treatment***					
Boiling	20 (19.4%)	-	**-**	1.14 (0.36–3.67), p = 0.822	**-**
Chlorine/Bleach	62 (60.2%)	**Reference**	**Reference**	**Reference**	**Reference**
Others	21 (20.4%)	-	-	2.81 (1.36–5.77), p = 0.005*	-
***Type of* latrine facility**					
Improved	143 (30.2%)	**Reference**	**Reference**	**Reference**	**Reference**
Unimproved	225 (47.5%)	0.75 (0.21–2.73), p = 0.664	0.77 (0.19–3.02), p = 0.703	0.83 (0.50–1.38), p = 0.470	1.47 (0.81–2.69), p = 0.208
***Household assets***					
Electricity	66 (13.9%)	1.73 (0.35–8.57), p = 0.500	2.12 (0.40–11.26), p = 0.377	0.58 (0.25–1.36), p = 0.212	1.38 (1.02–1.87), p = 0.034*
Radio	173 (36.5%)	1.96 (1.35–2.86), p<0.001*	2.21 (1.50–3.26), p<0.001*	1.00 (0.75–1.35), p = 0.983	1.37 (0.81–2.30), p = 0.238
Television	376 (79.3%)	0.57 (0.19–1.67), p = 0.302	0.67 (0.22–2.01), p = 0.474	0.68 (0.58–0.80), p<0.001*	0.98 (0.26–3.70), p = 0.977
Mobile phone	388 (81.9%)	0.50 (0.27–0.91), p = 0.023*	0.51 (0.28–0.90), p = 0.021*	1.11 (0.56–2.20), p = 0.776	1.57 (0.75–3.27), p = 0.230
Bank account	41 (8.7%)	0.48 (0.04–5.30), p = 0.553	0.55 (0.05–6.33), p = 0.635	0.77 (0.29–2.04), p = 0.600	1.26 (0.83–1.90), p = 0.280
Agricultural land	416 (87.8%)	0.50 (0.24–1.05), p = 0.066	0.55 (0.32–0.97), p = 0.037*	1.92 (0.47–7.88), p = 0.364	1.75 (0.62–4.96), p = 0.292
Cows and goats	227 (47.9%)	1.02 (0.31–3.34), p = 0.977	1.10 (0.34–3.62), p = 0.871	1.00 (0.71–1.42), p = 0.998	1.32 (0.52–3.31), p = 0.560
Chicken and ducks	364 (76.8%)	1.09 (0.52–2.33), p = 0.812	0.89 (0.45–1.76), p = 0.745	1.14 (0.38–3.45), p = 0.811	0.58 (0.39–0.86), p = 0.007*

* significant variables p<0.005

- variable omitted due to no/insufficient observations

^$^ Four participants declined to do pregnancy test. Pregnancy test was carried out using pregnancy test strips

### WASH factors among the study participants

Mostly, sources of water for drinking and household use 342 (72.2%) and 364 (76.8%) respectively, were not improved. Majority of the households using unimproved drinking water sources were from Magarini sub-County at 146 (74.1%) followed by Rabai sub-county at 196 (70.8%). The average time taken for one round trip to the water source was 52.2 minutes (SD: 41.8 minutes; range: 1–190). In Rabai sub-county, majority of the participants (181 (65.3%)) took between 30 and 60 minutes for one round trip to the water source, 50 (18.1%) took less than 30 minutes and 46 women (16.6%) took more than one hour to and from the water source. In Magarini sub-county, 86 women (43.7%) took between 30 and 60 minutes, 57 (28.9%) took less than 30 minutes and 54 (27.4%) women took more than one hour for one round trip. Only 103 (21.7%) participants treated their water to make it safer for drinking either through chlorine/bleach 62 (60.2%), or by boiling 20 (19.4%) and other methods 21 (20.4%) (Table 5).

The frequency of handwashing at critical times was reported; majority of the women always washed their hands after handling children faeces, before handling food and after toilet use 415 (87.6%), 394 (83.1%) and 462 (97.5%) respectively. The most reported anal cleansing material was water 349 (73.6%), followed by leaves/vegetation 74 (15.6%), toilet paper 36 (7.6%) and newspaper 15 (3.2%). In Rabai sub-county, majority of the women used water for anal cleansing 262 (94.5%), 9 (3.3%) used leaves/vegetation, 3 (1.1%) used newspaper/toilet paper. In Magarini Sub-County, 87 (44.2%) used water for anal cleaning, 65(32.9%) used leaves/vegetation, 33 (16.8%) used toilet paper and 12 (6.1%) used newspapers (Table 5).

The study participants had knowledge on the source of infection with S. *haematobium* and that the disease could affect the reproductive organs and would be good for one to get treated when infected. Some of the quotes from the study participants are listed below;

*P4; Yes*, *the problem is not the river but the dirty water*. *It is true the children are at risk*, *but the women are more at risk because if you get infected with bilharzia as a woman it will affect your reproductive organs and become prone to other diseases*. *It can damage the reproductive organs*.

P3; “Another way is when we swim in a dam, where someone who has bilharzia urinated or defaecated in the water, if you go and swim there you can get the disease”

*P5; “We can also get because we usually go to fetch water from the river and the dams*, *so if you step on the water you can get infected because not everyone wears shoes”*

Most study participants reported poor health seeking behaviour by community members which might be increasing the spread of the disease. Some of the reasons given were that they don’t like seeking medical attention because they are afraid of being tested for other diseases like HIV/AIDs.

*“P3; Some people don’t like*. *They have not accepted*. *I don’t know why they don’t like going to the hospital”*

“P1; Yes, here in our community that is how people behave, they think that they will be tested for HIV/AIDs, they are afraid”

“P9; Some go without being treated. At last they will be advised to seek for treatment. From the family and they will agree to go to the hospital”

*“P9; The men here when they become sick they keep to themselves but for the women we usually go to the hospital”*

The households that had improved toilet/latrine facilities were 143 (30.2%) while majority 225 (47.5%) had unimproved facilities. In Magarini sub-county, 61 (30.9%) households used improved facilities, 42 (21.3%) used unimproved facilities and 94 (47.7%) did not use any toilet/latrine facility. In Rabai Sub-county, 82 (29.6%) households used improved facilities, 183 (66.1%) used unimproved facilities and 12 (4.3%) did not use any toilet/latrine facility. Majority of the households 348 (73.4%) shared the toilet/latrine facility with other households.

### Univariable analysis of risk factors

Univariable analysis was performed for any STH, hookworm, *S*. *haematobium* and malaria infections. For any STH infection, variables associated with an increased odds of infection were; Rabai sub-county (OR = 8.43, p = 0.006), secondary level of education (OR = 2.69, p = 0.032) and casual laborer (OR = 6.41, p = 0.045). In addition, other types of occupation (OR = 3.67, p<0.001), practicing Islam (OR = 2.48, p = 0.036), women who were aged above 25 years when they were first pregnant (OR = 4.84, p = 0.002) had increased odds of STH infection. While variables associated with decreased odds of infection were; participants who had two pregnancies in their lifetime (OR = 0.31, p = 0.044) and households that owned a radio (OR = 1.96, p<0.001).

For *S*. *haematobium* infection, increased odds of infections were associated with; an average household income of between KES 5,000 and 10,000 (OR = 1.90, p = 0.002), practising Islam (OR = 1.80, p = 0.035), and participants not attending ANC (OR = 3.64, p = 0.014). While reduced odds of infections were associated with; participants who were housewives (OR = 0.47, p = 0.023), practised other occupations (OR = 0.23, p = 0.006), had two pregnancies in their lifetime (OR = 0.42, p = 0.047), participants taking anti-malarial tablets (OR = 0.59, p = 0.010) and participants who owned a television (OR = 0.68, p<0.001).

In the qualitative analysis, majority of the study participants felt that their source of water for drinking and domestic use could be the possible source of infection as the quotes below.

*P1; “For example*, *us women like going to wash clothes in the river and when we finish we also bathe there*. *When you go with the small kids you fetch water and put in a basin and leave them to play with it so that you can finish your work*, *which risks them getting infected”*

P1;” We have people who go to fetch water without a contain to draw the water so they will step in the water, so when other people come and fetch the same water and take it at home and drink without boiling it will affect them”

To control and prevent the diseases, the recommendations from the study participants were seeking health care if infected and preventing the children from swimming in infected waters which might not be possible due to their play habits as the quotes listed below: -

P3; “Treating drinking water and also not allowing the children to play in the dirty water”

p6; “I would like to get drugs here if I am infected with bilharzia than being told to go and buy elsewhere”

P8;” We should not allow our children to play in dirt water and when they are walking they should put on shoes so that they don’t step on areas where may be there those worms that cause bilharzia”

p8; “We should be tested at intervals because we are using the same water to know if we have the infection”

Nevertheless, there were misconception among some of the study participants of *S*. *haematobium* being a sexually transmitted infection (STI) as the quotes listed below;

P6; “I don’t see the difference between bilharzia and sexually transmitted diseases, because if you have bilharzia you are passing blood and pus just like the sexually transmitted diseases”.

P10; “When you are passing blood in the urine then at some point pus, that pus can cause those diseases like gonorrhoea”

P2; “If you are a lady and you have several sexual partners you risk getting the disease”

P6; “A husband can bring bilharzia to the woman because if they have sexual intercourse with another woman out there, they will bring it to the wife back at home”

*P7; “If your husband goes to have sexual intercourse with another woman out there he can come to infect you”*

For malaria infection, increased odds of infection were associated with; primary education (OR = 1.91, p = 0.027), average household income of above KES 10,000 (OR = 1.57, p = 0.014), participants with less than 5 live births in their lifetime (OR = 8.47, p = 0.026), those receiving ANC from other facilities compared to government facilities (OR = 5.50, p = 0.048). While reduced infection was associated with Rabai sub-county (OR = 0.19, p = 0.011), participants aged between 31 and 40 years (OR = 0.50, p = 0.049), participants practicing Islam (OR = 0.11, p = 0.006), participants with two or more than two pregnancies in their lifetime (OR = 0.27,p = 0.035), and (OR = 0.45,p = 0.007) respectively, and participants taking antimalarial tablets (OR = 0.12, p<0.001) (Table 5).

### Multivariable analysis of risk factors

There were significant increased odds of any STH infections among participants in Rabai sub-county, (aOR = 9.74; p = 0.026), women who were in business (aOR = 5.25; p<0.001), housewives/no job (aOR = 2.78; p = 0.003), were casual laborers (aOR = 27.03; p<0.001), and participants who owned a radio (aOR = 2.23; p = 0.012). Lower odds of any STH infections were observed among women who had two pregnancies in their lifetime (aOR = 0.25; p<0.001) (Table 6). Similarly, increased odds of hookworm infections were observed among women who were in business (aOR = 22.89; p = 0.004), housewives (aOR = 8.50; p = 0.029), casual laborers (aOR = 125.43; p = 0.006), and participants who owned a radio (aOR = 3.01; p = 0.002). Reduced odds of hookworm infection were observed among women who had two pregnancies in their lifetime (aOR = 0.14; p<0.001) and among women who owned agricultural land (aOR = 0.17; p = 0.033) (Table 6). Women who reported not attending ANC had increased odds of *S*. *haematobium* infections, (aOR = 7.21; p = 0.049) while those who were housewives had significantly lower odds of infection (aOR = 0.20; p = 0.005) (Table 6). For malaria infection, none of the factors showed increased or reduced odds of infection.

**Table 6:** Multivariable analysis of factors associated with any STH, hookworm, schistosomiasis and malaria infections.

Factors	Adjusted Odds Ratio (aOR); p-value			
	Any STH	Hookworm	*S*. *haematobium*	Malaria
**Demographics**				
***Sub-counties***				
Magarini	**Reference**	**Reference**		**Reference**
Rabai	9.74 (1.31–72.24); p = 0.026*	17.88 (0.61–523.08); p = 0.094		0.00 (0.00–5.64); p = 0.129
***Age groups***				
20 years and below				**Reference**
21–30				0.05 (0.00–19.39); p = 0.325
31–40				0.01 (0.00–11.39); p = 0.207
41–50				**-**
***Average income (Ksh)***				
Below 5000			**Reference**	**Reference**
Between 5000–10000			1.99 (0.77–5.10); p = 0.154	1.30 (0.01–115.27); p = 0.909
Above 10000			0.72 (0.03–14.84); p = 0.829	0.03 (0.00–2.91); p = 0.134
***Level of education***				
None	**Reference**			
Primary level	0.66 (0.32–1.36); p = 0.257			0.05 (0.00–31.19); p = 0.369
Secondary level	0.75 (0.51–1.11); p = 0.147			-
Post-secondary	-			-
***Occupation***				
Farmer	**Reference**	**Reference**	**Reference**	
Business	5.25 (2.58–10.67); p<0.001*	22.89 (2.76–189.66); p = 0.004*	-	
Housewife/No job	2.78 (1.41–5.47); p = 0.003*	8.50 (1.24–58.41); p = 0.029*	0.20 (0.06–0.61); p = 0.005*	
Salaried worker	3.58 (0.24–52.78); p = 0.354	6.02 (0.47–77.92); p = 0.169	**-**	
Casual laborer	27.03 (7.12–102.59); p<0.001*	125.43 (4.08–3856.36); p = 0.006*	0.24 (0.02–2.23); p = 0.208	
Others	24.15 (9.56–60.99); p<0.001*	120.97 (6.55–2234.21); p = 0.001*	**-**	
***Religion***				
Christian	**Reference**	**Reference**	**Reference**	**Reference**
Islam	1.53 (0.40–5.84); p = 0.538	2.61 (0.39–17.61); p = 0.325	0.81 (0.62–1.07); p = 0.136	-
Non practicing	5.13 (0.31–84.12); p = 0.252	17.45 (0.35–859.32); p = 0.150	0.72 (0.09–5.86); p = 0.762	2.22 (0.10–48.18); p = 0.612
**Pregnancy factors**				
***Number of live births in lifetime***				
None				**-**
<5				**Reference**
5 and above				54.70 (0.11–273.26); p = 0.207
***Age when first pregnant (years)***				
<18 years	**Reference**	**Reference**		
18–25	0.29 (0.07–1.19); p = 0.086	0.19 (0.03–1.22); p = 0.079		
>25	1.94 (0.65–5.77); p = 0.235	2.47 (0.73–8.44); p = 0.148		
***Number of pregnancies in lifetime***				
One	**Reference**	**Reference**	**Reference**	**Reference**
Two	0.25 (0.20–0.32); p<0.001*	0.14 (0.09–0.23); p<0.001*	0.10 (0.00–45.62); p = 0.465	0.40 (0.10–48.18); p = 0.614
More than two	0.64 (0.22–1.85); p = 0.412	0.39 (0.15–1.07); p = 0.067	0.81 (0.02–40.90); p = 0.917	-
***Place of receiving ANC***				
Government health facility			**Reference**	**Reference**
Other			2.10 (0.11–39.41); p = 0.621	14.01 (0.4–225.00); p = 0.081
Not attending ANC			**7.21 (1.00–52.02); p = 0.049** *	1.01 (0.01–98.86); p = 0.996
***Given iron***				
Yes				**Reference**
No				2.76 (0.06–134.73); p = 0.609
***Taken antimalarial tablets***				
Yes			3.13 (0.79–12.36); p = 0.104	
No			**Reference**	
**Individual WASH factors**				
***Handwash before preparing food***				
Always		**Reference**		
Sometimes		1.20 (0.15–9.56); p = 0.864		
Never		1.19 (0.29–4.95); p = 0.813		
***Anal cleansing material used***				
Toilet paper				**Reference**
Water				0.15 (0.00–28.97); p = 0.478
Newspaper				-
Leaves/vegetation				0.15 (0.00–14.09); p = 0.411
***Household assets***				
Had a Radio vs did not	2.23 (1.19–4.16); p = 0.012*	3.01 (1.50–6.04); p = 0.002*		
Had a Mobile phone vs did not	0.39 (0.14–1.08); p = 0.070	0.31 (0.08–1.16); p = 0.082		
Had Agricultural land vs did not		0.17 (0.03–0.87); p = 0.033*		
Had Electricity vs did not				1.44 (0.02–85.76); p = 0.861
Had Chicken and ducks vs did not				5.56 (0.38–81.75); p = 0.211

*Indicates a statistically significant association

-variable omitted because of insufficient number of observations

## Discussion

Few studies have been conducted to quantify the extent of schistosomiasis, STH and malaria co-infections among WRA living in endemic regions of Kilifi. Data from this study has demonstrated that there was low to moderate prevalence of any STH infection at 5.2%, malaria 8.3% and *S*. *haematobium* at 22.3% among WRA. This is in contrast with previous studies conducted in September 2011 among adult community members in the neighboring Kwale County in the coastal region of Kenya which showed high prevalence of STH (41.7%) and moderate *S*. *haematobium* (18.2%) infection [15]. This could be attributed to over years, most WRA visiting ANCs in the country have been receiving treatment for STH with albendazole which could be contributing to low STH infection. More so, the MoH Kenya has been conducting Lymphatic filariasis MDAs in Kenya according WHO 2017 recommendations, whereby part of the treatment regime is albendazole, hence community members have been receiving treatment in this area [34]. In addition, the school-based deworming program (SBDP) in the country could be a contributor to the reduced STH infection among WRA noting that SAC have been treated annually for over eight years. This could be responsible for the reduced re-infections in the community setting [35].

The moderate *S*. *haematobium* infections could be attributed to the fact that the disease is localized unlike STH, control through treatment is mostly done after diagnosis with the parasitic infection and availability of the drugs. A similar study done in Kwale County reported low prevalence of infections with the parasitic diseases [17]. In addition, schistosomiasis is a water borne disease which requires many control strategies including WASH and behavior change by community members for effective control. Notably, these factors might have resulted in the observed high prevalence of schistosomiasis which is still above the newly-defined WHO target of <1% [36]. From the qualitative data, the study participants felt that the three parasitic diseases were common in the area and had knowledge of the signs and symptoms of the diseases. Another study conducted by Masaku *et al* in the same county on parents of PSAC showed that they knew about the cause of the diseases, signs and symptoms and some control measures [37].

### Malaria

Our findings also show that malaria is endemic in the area although at low prevalence. This gives a double burden to the WRA especially during pregnancy. We suggest that efforts should be made to integrate malaria programmes with specific NTDs occurring in this area. Another study done in the area in 2014 showed malaria was declining but still prevalent among women attending prenatal care [38]. This could be associated with failure to realize that they need to protect themselves from malaria by sleeping under a treated net and seeking health care in case one is ill. A study done by Nkem and colleagues showed that high levels of education were closely associated with improved knowledge and practice about the appropriate strategies for the prevention and treatment of malaria [39]. Similar studies done in Colombia showed that respondents in high risk areas did nothing to prevent malaria transmission outdoors with self-medication, poor adherence to treatment, as well as lack of both indoor and outdoor vector control measures being significantly associated with higher malaria risk [40]. Also, those women who sought for ANC services in other facilities other than the government facilities had increased odds of malaria infection. This could be attributed to the availability of free insecticide treated nets (ITNs) and free malaria treatment in Kenyan government facilities as opposed to other facilities.

### STH

Casual laborer, owning a radio and having no job were significantly associated with STH infection. Many studies have associated low income and poverty to be closely associated with STH infection. Hierarchical multinomial models in Brazil demonstrated that relative socio-economic status was significantly associated with STH and schistosomiasis co-infection [41]. For the qualitative analysis, study participants felt that low socio-economic status is one of the reasons some people do not own pit latrines due to the cost of building materials. A study done by Oswald *et al*., 2019 showed that sanitation coverage in schools and villages had a general impact on likelihood of helminths infection among the communities [42]. Another similar study conducted in Timor-Leste by Campbell *et al* showed that WASH factors were key determinants of helminths infection [43]. Secondary level of education was significantly associated with any STH infection. Other similar studies done elsewhere have also shown significant association between level of education and STH infection [4]. This could be attributed to poor sanitation and personal hygiene whereby the target population tend to expose themselves to infection by not washing hands frequently. Especially before eating and after relieving oneself probably due to inadequate water supply considering that the study area is a rural setting with no piped water. Notably, data on water source was not captured in this study. Religious belief (practicing Islam) was also associated with any STH infection. A similar study done in Ghana, West Africa, also reported religion to be a significant factor (p = 0.0181) influencing STH infection [44]. Geographical location of Rabai sub-county was significantly associated with any STH. Other studies done elsewhere have shown association between geophagy and STH infection which could be associated with the significance difference in the two sub-counties (Rabai and Magarini) [45, 46]. Women who were aged above 25 years when they first got pregnant had increased odds of STH infection. Separate studies done in Ethiopia and Thailand have found mothers to be highly infected with STH which was associated with maternal anaemia, in particular in the event of late ANC enrolment [47, 48]. This calls for enhanced screening and treatment for mothers visiting ANC considering that anemia causes craving for geophagy which in turn exposes mothers to STH infection.

### Schistosomiasis

The results of this study found that there were increased odds for *S*. *haematobium* infection among WRA with average household income between 5,000 and 10,000 Kenya shillings. Previous similar study done in Yemen by Sady and colleagues has shown significant association between low household monthly income (*P*  =  0.003) and schistosomiasis infection [49]. This could be due to women with average income exposing themselves to schistosomiasis infection while fetching water or doing domestic chores in infested water bodies. Participants not attending antenatal care (ANC) had increased odds of *S*. *haematobium* infection. This could be attributed to poor knowledge and understating of possible risk factors and control measures. Those WRA who visit ANC are usually educated on health matters especially diseases that are endemic in their specific locality and at times they get tested and treated for these parasitic diseases free of charge. In a study done in Nigeria, it was shown that literacy on the family head was a protector to schistosomiasis infection and that the infection was closely associated with poverty [50]. While for the qualitative analysis, poor health seeking behavior for the parasitic diseases was mentioned by the study participants to be a common habit due to fear of being tested for other diseases. This calls for enhanced health promotion to educate the local community members on the importance of seeking healthcare when they are sick for improved control and prevention of the parasitic diseases. In addition, qualitative results found out that there were misconceptions on *S*. *haematobium* being a STI. A similar study done in Ethiopia by Asseffa and colleagues showed that the community members had misconceptions about schistosomiasis on the mode of infection [51]. There were increased odds of malaria infection among WRA with primary education.

### Study limitations

One of the study limitations was that we used the WHO recommended Kato Katz technique which can miss some egg counts in an area with low intensity of infection especially after several rounds of MDA. We recommend the need for more sensitive diagnostic techniques like MC Master for programmatic monitoring for STH in future [52]. On the other hand, the results cannot be generalizable for the qualitative arm of the study to other parts of the country as the study was exploring the factors that have contributed to slow decline of STH infection in specific counties. Further, the other arm of the study was exploring the factors that have contributed to co-infections of helminthiasis and malaria in Kilifi County. Other limitations of the study were lack of structured observations, failure to triangulate secondary data, not measuring compliance and logistical issues of the MDA which could have improved the study outcomes. In addition, lack of structured observations of water supply, sanitation infrastructure and associated behaviors was a major limitation to the study due to limited funding.

## Conclusions

This study demonstrated that STH, *S*. *haematobium* and malaria are still a public health problem to WRA. The occurrence of co-infection was low and was recorded between any STH and *S*. *haematobium* and between *S*. *haematobium* and malaria. Due to the observed cases of schistosomiasis, treatment in WRA should be initiated in the study area and conducted as per the current WHO guidelines. Among the pregnant women, prevalence of malaria was highest as compared to non-pregnant women, therefore, malaria control interventions should be intensified among this risk group in Kilifi County. Some of the associated risks of infection were geographical location, women not attending ANC, socio-economic and WASH factors. Hence the need to implement integrated control efforts of STH, *S*. *haematobium* and malaria by inclusion of WASH programmes, health promotion to demystify the misconceptions, awareness creation on behaviour change communication (BCC) and the need to visit antenatal care.

## Abbreviations

STH: Soil-Transmitted Helminths
NTDs: Neglected Tropical Diseases
SAC: School-Age Children
WRA: Women of Reproductive age
WHO: World Health Organization
MDA: Mass Drug Adminstration
MoH: Ministry of Health
NSBDP: National School-Based Deworming Programme
WASH: Water Sanitation and Hygiene
ALB: albendazole
epg: Eggs Per Gram
ODK: Open Data Kit
SD: Standard Deviation
CI: Confidence Intervals
OR: odds ratio
SERU: Scientific and Ethics Review Unit
KEMRI: Kenya Medical Research Institute
